# Mechanical Dyssynchrony Combined with Septal Scarring Reliably Identifies Responders to Cardiac Resynchronization Therapy

**DOI:** 10.3390/jcm12186108

**Published:** 2023-09-21

**Authors:** Jürgen Duchenne, Camilla K. Larsen, Marta Cvijic, Elena Galli, John M. Aalen, Boudewijn Klop, Oana Mirea, Alexis Puvrez, Stéphanie Bézy, Laurine Wouters, Lennert Minten, Per A. Sirnes, Faraz H. Khan, Gabor Voros, Rik Willems, Martin Penicka, Erik Kongsgård, Einar Hopp, Jan Bogaert, Otto A. Smiseth, Erwan Donal, Jens-Uwe Voigt

**Affiliations:** 1 Department of Cardiovascular Sciences, KU Leuven, 3000 Leuven, Belgiumlennert.minten@kuleuven.be (L.M.);; 2 Department of Cardiovascular Diseases, University Hospitals Leuven, 3000 Leuven, Belgium; 3 Institute for Surgical Research, Oslo University Hospital and University of Oslo, 0450 Oslo, Norway; 4 Institute of Clinical Medicine, University of Oslo, 0313 Oslo, Norway; 5 Department of Cardiology, Oslo University Hospital, 0379 Oslo, Norway; 6 Inserm, LTSI-UMR, 1099, 35042 Rennes, France; elena.galli@chu-rennes.fr (E.G.);; 7 Department of Cardiology, CHU Rennes, 35033 Rennes, France; 8 Department of Cardiology, University of Medicine and Pharmacy, 200349 Craiova, Romania; 9 Cardiovascular Center Aalst, OLV Clinic, 9300 Aalst, Belgium; 10 Division of Radiology and Nuclear Medicine, Oslo University Hospital, 0379 Oslo, Norway; 11 Department of Imaging and Pathology, KU Leuven, 3000 Leuven, Belgium; 12 Department of Radiology, University Hospitals Leuven, 3000 Leuven, Belgium

**Keywords:** apical rocking, cardiac resynchronization therapy, dyssynchrony, myocardial scar, myocardial work, reverse remodelling, septal flash, systolic stretch index

## Abstract

**Background and aim:** The presence of mechanical dyssynchrony on echocardiography is associated with reverse remodelling and decreased mortality after cardiac resynchronization therapy (CRT). Contrarily, myocardial scar reduces the effect of CRT. This study investigated how well a combined assessment of different markers of mechanical dyssynchrony and scarring identifies CRT responders. **Methods:** In a prospective multicentre study of 170 CRT recipients, septal flash (SF), apical rocking (ApRock), systolic stretch index (SSI), and lateral-to-septal (LW-S) work differences were assessed using echocardiography. Myocardial scarring was quantified using cardiac magnetic resonance imaging (CMR) or excluded based on a coronary angiogram and clinical history. The primary endpoint was a CRT response, defined as a ≥15% reduction in LV end-systolic volume 12 months after implantation. The secondary endpoint was time-to-death. **Results:** The combined assessment of mechanical dyssynchrony and septal scarring showed AUCs ranging between 0.81 (95%CI: 0.74–0.88) and 0.86 (95%CI: 0.79–0.91) for predicting a CRT response, without significant differences between the markers, but significantly higher than mechanical dyssynchrony alone. QRS morphology, QRS duration, and LV ejection fraction were not superior in their prediction. Predictive power was similar in the subgroups of patients with ischemic cardiomyopathy. The combined assessments significantly predicted all-cause mortality at 44 ± 13 months after CRT with a hazard ratio ranging from 0.28 (95%CI: 0.12–0.67) to 0.20 (95%CI: 0.08–0.49). **Conclusions:** The combined assessment of mechanical dyssynchrony and septal scarring identified CRT responders with high predictive power. Both visual and quantitative markers were highly feasible and demonstrated similar results. This work demonstrates the value of imaging LV mechanics and scarring in CRT candidates, which can already be achieved in a clinical routine.

## 1. Introduction

Cardiac resynchronization therapy (CRT) is indicated in patients with symptomatic heart failure, reduced left ventricular (LV) ejection fraction (LVEF ≤ 35%), and a wide QRS (≥130 ms), preferably with a left bundle branch block (LBBB) morphology. Despite technical improvements and accumulating experience, the non-response rate to CRT remains high (circa 30–40%) [1]. On the other hand, patients who would benefit from CRT are denied therapy by current selection criteria [2]. More than a decade ago, the assessment of mechanical dyssynchrony using echocardiography was proposed to improve patient selection for CRT. However, its use has been refuted due to disappointing results from one observational and two randomized studies [3,4,5]. The proposed parameters were neither sensitive nor specific enough to reduce non-response [4] and lead to unnecessary pacing and increased mortality in patients [5].

Since then, a better understanding of the pathophysiology has led to the identification of motion patterns that truly reflect a curable substrate for CRT [6,7,8,9,10,11]. Among those, both visual markers such as septal flash (SF) and apical rocking (ApRock), as well as quantitative markers such as the systolic stretch index (SSI) and lateral-to-septal (LW-S) work difference, have demonstrated to be specific, easy to use, outperforming QRS duration and morphology, and to be associated with favourable long-term survival after CRT [9,11,12,13].

In contrast, both the extent and location of myocardial scar influence myocardial motion patterns and have a negative impact on CRT outcome [11,14]. Recent studies have therefore tried to integrate the assessment of scar with mechanical dyssynchrony—either LW-S work difference [11] or SF/ApRock [15]. However, a direct comparison between the performance of different parameters of mechanical dyssynchrony and the impact of integrating information on myocardial scarring on their prediction of CRT response has not yet been performed.

The present prospective multicentre study investigated the impact of myocardial scarring on the prediction of a CRT response using an echocardiographic evaluation of mechanical dyssynchrony. Cardiac magnetic resonance imaging (CMR) with late gadolinium enhancement (LGE) was used to assess myocardial scarring. The primary endpoint was volumetric reverse remodelling at 12 months follow-up. The secondary endpoint was long-term survival. The combined approach of integrating information of myocardial scarring with mechanical dyssynchrony was: (I) compared among both visual (SF/ApRock) and quantitative (SSI/LW-S work difference) markers, (II) evaluated for its additive predictive value over those markers alone, and (III) tested for its added value over LV ejection fraction, QRS duration and morphology.

## 2. Methods

### 2.1. Study Population

A total of 200 heart failure patients referred for CRT were prospectively recruited through the cardiology departments of the University Hospitals Leuven, Belgium; Oslo University Hospital, Norway; CHU Rennes, France; and OLV Hospital Aalst, Belgium between August 2015 and November 2017. The patients were all-comers with an indication for CRT, according to the European Society of Cardiology guidelines, at the time of inclusion [16]. Exclusion criteria were recent myocardial infarction or cardiac surgery (≤12 months) and severe aortic stenosis.

### 2.2. Echocardiography: Conventional Parameters

Echocardiography was performed within 1 week before and 12 months after CRT implantation, using Vivid E9 or E95 ultrasound scanners (GE Vingmed Ultrasound, Horten, Norway). Blood pressure was measured using the brachial cuff method at the beginning of the examination. LV volumes and LVEF were measured using the modified Simpson’s biplane method. Regional and global longitudinal strains (GLSs) were measured by speckle tracking (frame rate > 60 frames/s) using the 18-segment model. All analyses were performed offline using EchoPAC version 202 (GE Vingmed).

### 2.3. Mechanical Dyssynchrony: Visual Markers

#### 2.3.1. Septal Flash

Visual assessment of SF was performed in the apical four- and three-chamber view. SF was defined as a rapid inward motion and shortening of the (antero-)septal wall, occurring directly after the onset of QRS and before shortening of the lateral wall (Graphical Abstract). Presence of SF was recorded if it was seen in either the four- or three-chamber view, or both.

#### 2.3.2. Apical Rocking

Visual assessment of ApRock was performed in the apical four- and three-chamber view. ApRock was defined as a systolic septal-to-lateral motion sequence of the apical myocardium (Graphical Abstract). The laterally directed component should occur during ejection time, i.e., before mitral valve opening. The presence of ApRock was recorded if it was seen in either the four- or three-chamber view, or both.

#### 2.3.3. Decision-Making for Septal Flash and Apical Rocking

Three readers in Leuven (JD, MC, JUV) performed independent visual readings of SF and ApRock prior to CRT implantation. At the time of the reading, these observers were blinded to all patient information. Mechanical dyssynchrony was considered present when either SF or ApRock, or both, were observed. For further analysis, the majority opinion of the three readers was used.

### 2.4. Mechanical Dyssynchrony: Quantitative Markers

#### 2.4.1. Systolic Stretch Index

SSI was calculated from longitudinal strain traces of the four-chamber view as the sum of the septal systolic stretch and lateral systolic pre-stretch before aortic valve closure [9] (Graphical Abstract). Values from the basal and mid-ventricular segments were averaged to represent respective walls. Mechanical dyssynchrony was considered present when SSI was ≥3.1% [9].

#### 2.4.2. Lateral-to-Septal Work Difference

Regional systolic work [17] in the septum and lateral wall was calculated as the average work from the respective basal- and mid-ventricular segments in the apical four-chamber view, as previously described [11] (Graphical Abstract). The difference between work in the lateral wall and septum (LW-S work difference) was used as a measure of asymmetry in myocardial loading. Mechanical dyssynchrony was considered present if the LW-S work difference was ≥860 mmHg% [11]. Strain and work analyses were performed by one observer in Oslo (JMA) blind to all patient information.

### 2.5. Myocardial Scarring

Prior to CRT implantation, patients were scanned with a 1.5 or 3.0 Tesla MRI unit (Aera or Verio, Siemens Healthcare, Erlangen, Germany; Ingenia, Philips Healthcare, Best, The Netherlands; Signa HGXT, GE Healthcare, Chicago, USA). LGE images were obtained as previously described [18]. Scar size was quantified semi-automatically using Segment software v2.0 R5270 [19] by an experienced observer blind to other patient data (CKL). The origin of LGE in the septum was determined as ischemic or non-ischemic. Myocardial scarring was assessed using a 17-segment model and reported regionally as percentage of total amount of scarred tissue per LV wall: anterior, septal (antero-septal and septal), inferior, and lateral (lateral and posterior). The apical cap was excluded.

LGE-CMR was obtained in 125 of 200 patients. The main reasons for the other 75 patients not undergoing LGE-CMR were non-CMR-compatible devices (*n* = 42) and severely reduced renal function (*n* = 11). For the remaining patients (*n* = 22), reasons included claustrophobia, intracranial metal implants, and logistic causes.

In patients without LGE-CMR (75 of 200), the presence of large myocardial scarring was excluded based on all of the following: (I) no evidence of a previous myocardial infarction in the medical history, laboratory values, ECG, or scintigraphy; (II) no relevant stenosis on a recent coronary angiogram (max. 1 year prior to CRT implantation); and (III) no history of coronary intervention (percutaneous or surgical). In 30 of 75 patients without CMR data, scarring could not be ruled out. These patients were excluded.

### 2.6. Cardiac Resynchronization Therapy

Patients underwent standard implantation of a biventricular pacing system. Coronary venography was used to optimize the placement of the LV lead in a lateral or posterolateral vein. The device was programmed in a conventional biventricular pacing mode and retested prior to hospital discharge.

### 2.7. Endpoints

Primary endpoint was CRT response, defined as a ≥15% reduction in LV end-systolic volume at 12 months follow-up. All volumes were measured independently in three different centres (Leuven, Oslo, and Rennes) by readers blinded to any other data (BK, PAS, EG). Reported volumes are the averages of the three readings. Volumetric CRT response (yes/no) was determined based on a majority decision.

Secondary endpoint was time-to-death of any cause, heart transplant, or LV assist device implantation, with an average follow-up time of 44 ± 13 months after CRT implantation (median 1543 (IQR 139–1726) days).

### 2.8. Statistical Analysis

Analysis was performed using SPSS Statistics 20 (IBM, Chicago, IL, USA) and MedCalc 15 (MedCalc, Ostend, Belgium). All continuous variables are expressed as mean ± standard deviation if normally distributed, otherwise by median ± interquartile range. Normality was assessed using the Shapiro–Wilk test. (Un-)paired sample *t*-tests or chi-squared tests were used for comparison of continuous or categorical variables, respectively. Univariate or multivariate linear regression analysis was used to identify predictors of reverse remodelling. Collinearity was tested using the variance inflation factor (VIF). Receiver operating characteristics (ROC) curves with area under the curve (AUC) values were used to determine the discriminative ability. To combine the assessment of two or more parameters, logistic regression was used to calculate a linear combination of parameters, the equation of which was then used for ROC curves. Nagelkerke’s R^2^ was reported for the predictive value of multivariate logistic regression models. The Delong method was used to compare ROC curves. Survival data are presented as hazard ratios (HR) (Cox regression) and Kaplan–Meier curves with a log-rank test for comparison. Inter-rater variability was tested using intra-class correlation (ICC) by two readers blinded to any other data (original reader and OM). Statistical significance was set at a two-tailed probability level of *p* < 0.05. Missing data were handled using pairwise deletion per parameter.

## 3. Results

Of the 170 included patients, 2 patients had died and 1 underwent heart transplantation, before 12 months follow-up. These three patients were considered as non-responders. The primary endpoint of the ≥15% reduction in LV ESV was achieved in 121 patients (72%). Among non-responders were more patients with ischaemic cardiomyopathy (Table 1). At follow-up, LV volumes, LVEF, and GLS improved substantially in responders, in contrast to non-responders (Table 2).

### 3.1. Predictive Value of Markers of Mechanical Dyssynchrony

Prior to CRT, 119 (70%) patients showed either SF or ApRock, 117 (69%) patients had an SSI ≥ 3.1%, and 115 (68%) patients demonstrated LW-S work asymmetry ≥ 860 mmHg·% (Table S1). These markers were observed more frequently among responders compared to non-responders (all *p* < 0.0001). Regression analysis revealed a direct relation between the presence of SF or ApRock (R = 0.51), SSI (R = 0.46), and LW-S work difference (R = 0.41) vs. reverse remodelling (all *p* < 0.0001).

**Figure 1:**
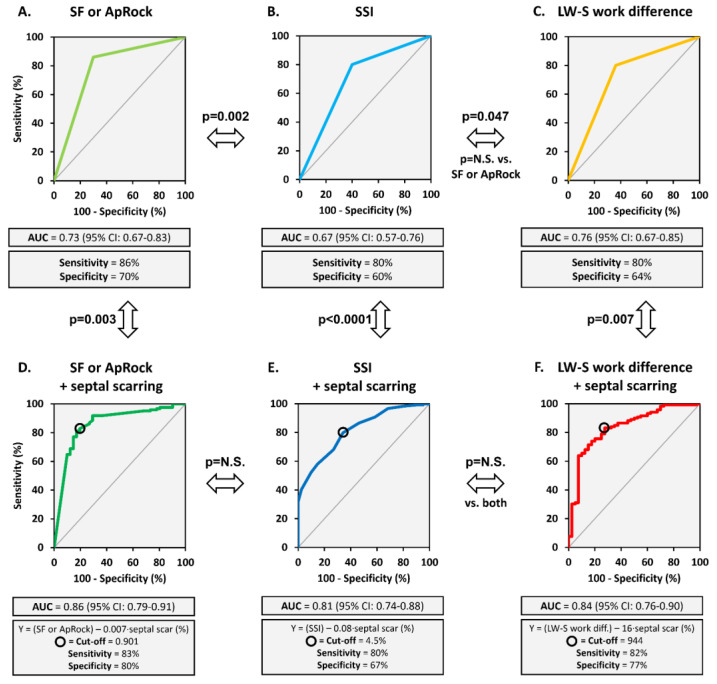
Presence of mechanical dyssynchrony, combined with septal scarring, identifies CRT response. ROC curve displaying as predictors of CRT response. Top row: markers, not combined with septal scarring info, (**A**) SF or Aprock, (**B**) SSI, and (**C**) LW-S work difference. Bottom row: markers combined with septal scarring info, (**D**) SF or ApRock + septal scarring, (**E**) SSI + septal scarring, and (**F**) LW-S work difference + septal scarring. Statistical differences between plots are indicated.

The AUCs were 0.73 for the presence of SF or ApRock, 0.67 for SSI, and 0.76 for LW-S work difference (all *p* < 0.0001) (Figure 1). The predictive power of SF or ApRock and LW-S work difference was not significantly different (*p* > 0.05), but both were higher compared to SSI (*p* = 0.002 and *p* = 0.047, respectively).

When analysing patients with LBBB only (*n* = 148), the AUCs were similar and ranged between 0.69 and 0.76 (Table S2).

**Figure 2:**
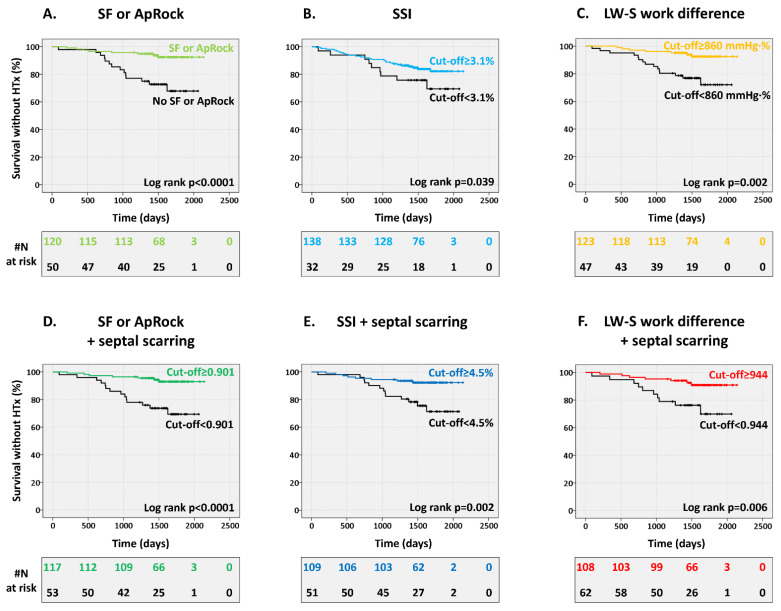
Presence of mechanical dyssynchrony, combined with septal viability, predicts survival without heart transplant (HTx). Kaplan–Meier curve stratified according to the presence of mechanical dyssynchrony. Top row: markers, not combined with septal scarring info, (**A**) SF or ApRock, (**B**) SSI, and (**C**) LW-S work difference. Bottom row: markers combined with septal scarring info, (**D**) SF or ApRock + septal scarring, (**E**) SSI + septal scarring, and (**F**) LW-S work difference + septal scarring.

**Figure 3:**
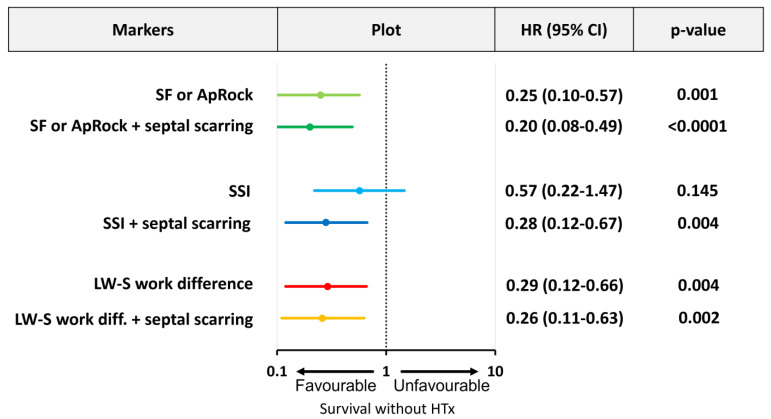
Mechanical dyssynchrony presence, combined with septal viability, reduces the hazard of death or heart transplant (HTx). Cox regression model with hazard-ratio (HR) for the presence of SF or ApRock (light green line, row 1) + septal scarring (dark green line, row 2), SSI (light blue line, row 3) + septal scarring (dark blue line, row 4), and LW-S work difference (red line, row 5) + septal scarring (yellow line, row 6).

The presence of SF or ApRock, SSI ≥ 3.1%, and LW-S work difference ≥ 860 mmHg·% were significant predictors of survival at long-term follow-up (Figure 2). The mortality rate was lower in patients where markers of mechanical dyssynchrony were present; 75% lower with the presence of SF or ApRock, 43% lower for SSI, and 61% lower for LW-S work difference (all, but SSI *p* < 0.05) (Figure 3). The echocardiographic assessment of SF or ApRock was feasible in all patients, SSI in 165 (97%) and work analysis in 166 (96%) patients.

### 3.2. Myocardial Scarring

In multivariate linear regressions, SF or ApRock, SSI, and LW-S work difference, together with ischemic/non-ischemic aetiology, but not QRS morphology (LBBB or non-LBBB) nor QRS duration or LVEF, were independent predictors of volumetric change after CRT (Table 3).

Scarring was present in 58 (34%) patients. In total, 43 patients (25%) had some degree of scar in the anterior wall, 54 (32%) in the septum, 52 (31%) in the inferior wall, and 36 (21%) in the lateral wall. Univariate linear regression showed an inverse correlation between the percentage of LV scarring and reverse remodelling (R = −0.43, *p* < 0.0001). Multivariate linear regression, including the percentage of anterior, septal, inferior, and lateral wall scarring, revealed that only septal scarring was a significant predictor of reverse remodelling (Table 4). Furthermore, the absence of septal wall scarring independently predicted a response with an AUC of 0.67 (95% CI: 0.52–0.83) (*p* < 0.05).

### 3.3. Combining Mechanical Dyssynchrony and Septal Scarring

In multivariate linear regressions, only markers of mechanical dyssynchrony and septal scarring remained independent predictors of reverse remodelling after CRT (Table 5) (all *p* < 0.05). QRS morphology, QRS duration, and LVEF were not predictive (all *p* > 0.05).

The AUCs for the combined assessment of the markers of mechanical dyssynchrony and septal scarring as predictors of CRT response were 0.86 for the presence of SF or ApRock, 0.81 for SSI, and 0.84 for LW-S work difference (all *p* < 0.0001) (Figure 1). While the predictive power for CRT response of the different combined approaches did not differ significantly among each other (*p* > 0.05 for all), all AUCs were significantly better than the markers of mechanical dyssynchrony alone (*p* < 0.01 for all). When analysing patients with LBBB only (*n* = 148), the AUCs were similar and ranged between 0.82 and 0.85 (Table S2).

The combined approaches were strong and significant predictors and the reduced risk of death at 44 months follow-up (Figure 2). Furthermore, adding information on septal scarring improved the mortality rate; 80% lower for SF or ApRock, 72% lower for SSI, and 74% lower for LW-S work difference (all *p* < 0.01) (Figure 3).

### 3.4. Ischemic vs. Non-Ischemic Patients

The predictive power of the combined assessments for a CRT response was similar in the subgroups of patients with and without ischaemic aetiology of cardiomyopathy (Table 6). AUCs ranged from 0.75 to 0.80 for patients with ischemic heart disease, while patients with non-ischemic heart disease showed an AUC range between 0.79 and 0.87.

### 3.5. Reproducibility

The average ICC between the three centres for assessing the presence of reverse remodelling (delta ESV) at 12 months was 0.87 (95%CI: 0.81–0.91). Inter-rater agreement of the tested variables was good, with ICC = 0.73 (95%CI: 0.69–0.81) for SF or ApRock, ICC = 0.71 (95%CI: 0.62–0.78) for SSI, and ICC = 0.82 (95%CI: 0.76–0.87) for LW-S work.

## 4. Discussion

Data from this prospective, multicentre study demonstrate that combining the assessment of mechanical dyssynchrony using echocardiography with information on myocardial scarring improves the accurate identification of patients who will respond to CRT with reverse remodelling and who have better long-term survival after CRT. Septal wall scarring was shown to significantly affect CRT response predictions by mechanical dyssynchrony, as well as survival. The predictive power of a combined assessment, including information on septal scarring, was significantly stronger than that of mechanical dyssynchrony alone—independent of using either visual (SF/ApRock) or quantitative markers (SSI or LW-S work difference)—with no difference among the markers. Lastly, both the presence of mechanical dyssynchrony and septal scarring were stronger predictors of CRT responses when compared to LV ejection fraction, QRS duration, and morphology.

### 4.1. Influence of Myocardial Scarring on Mechanical Dyssynchrony and Response

SF, ApRock, SSI, and LW-S work differences are features specific to mechanical dyssynchrony in CRT candidates, which have already been shown to predict response and long-term survival after CRT in other prospective studies [9,11,13]. However, parameters of mechanical dyssynchrony are affected by regional scarring. Interestingly, in our study, lateral wall scarring was less predictive of CRT response than septal scarring. This can be explained by the reduction in the typical LBBB-induced septal motion pattern due to the decreased contractility of the lateral wall. The septum is thereby less stretched and SF and ApRock are diminished [18,20]. As a result, the information on lateral scarring is carried by the mechanical dyssynchrony parameters, and considering lateral scarring on top of an assessment of mechanical dyssynchrony has little added value.

In the present study, scarring in the septal wall proved to be the strongest scarring determinant of non-response after CRT, among the different LV regions, including the lateral wall. Notably, the deformation pattern of a scarred septum can be very similar to a remodelled and dyssynchronous septum in LBBB, which is dysfunctional but viable. In this scenario, information on septal scarring provides an added predictive value in a multivariate analysis as it helps to distinguish both situations [11,15]. As a result, the prediction of a CRT response becomes more specific without loss in sensitivity (Figure 1).

Furthermore, while the predictive power of the combined assessments towards CRT response appears similar between patients with or without ischemic heart disease, it provided a high sensitivity in non-ischemic patients (resulting in a low number of false negatives) and a high specificity in ischemic patients (resulting in a low number of false positives) (Table 6).

### 4.2. Comparison among Different Mechanics-Based Dyssynchrony Parameters

In the past years, evidence has accumulated that mechanical dyssynchrony is the fundamental pathophysiologic principle that leads to remodelling in conduction delays such as LBBB and that imaging dyssynchrony can better identify patients who could benefit from CRT. Besides the visual assessment of SF and ApRock, a multitude of quantitative parameters has been proposed, with two promising candidates being SSI [21] and LW-S work difference [11]. They all specifically consider the interaction between septal and lateral wall function. The advantage of quantitative parameters is a potentially lower user dependence, at the cost of the need for high-quality images and detailed, time-consuming regional deformation analysis. Myocardial work has the advantage of incorporating information on myocardial loading but requires more advanced post-processing. Our findings indicate that LW-S-work difference, as well as the visual analysis of SF and ApRock, have very comparable predictive values that both outperform SSI. However, when combined with information on septal scarring, all three approaches perform similarly well.

An important advantage of markers of mechanical dyssynchrony is their feasibility in clinical routine; they can be easily obtained either visually or by a routine speckle-tracking-based analysis, which is feasible in nearly all CRT candidates (≥96% of all patients in our study). Furthermore, the inter-rater agreement in our study was high.

### 4.3. Clinical Implications

The present multicentric prospective study adds to the evidence that assessment of mechanical dyssynchrony and scarring by imaging can reliably, and relatively simply, identify CRT candidates. Furthermore, it highlights that a better understanding of the mechanisms of dyssynchrony also allows a better clinical judgement. The presence of SF or ApRock can be determined easily on echocardiographic apical images in nearly all patients. SSI and LW-S work differences are based on speckle-tracking analysis, which has become clinical routine in recent years. Scar assessment by CMR is of course an additional burden. However, a majority of centres already perform LGE-CMR as a routine investigation prior to CRT to avoid LV lead placement in a scar.

### 4.4. Limitations

In a substantial number of patients, LGE-CMR is not feasible due to, e.g., severely reduced renal function or a previously implanted device. Wider use of renal-saving contrast agents and CMR-compatible devices will be helpful. The use of nuclear scintigraphy as an alternative option for assessing scarring is limited due to its high cost and patients’ exposure to ionizing radiation [22].

Ruling out myocardial scarring based on patient history and coronary angiogram has the limitation of disregarding (particularly) scarring of non-ischemic origin, which can also affect the CRT response [22].

Myocardial work analysis is currently only available from one vendor. Future developments could expand this methodology to other vendors.

Data on the LV lead position were not available and may have provided additional insights.

Even though evidence on the clinical use of mechanical dyssynchrony as a CRT indicator has already been demonstrated in multiple (observational) trials, solid evidence from randomized controlled trials is needed if imaging-based selection criteria are to be included as guideline indications [23].

## 5. Conclusions

The present study shows that readings of mechanical dyssynchrony—combined with an assessment of septal scarring—prior to CRT implantation have an added predictive value regarding reverse remodelling and long-term survival, compared to mechanical dyssynchrony alone. Patients with the presence of mechanical dyssynchrony on echocardiography—shown by either SF, ApRock, high SSI, and high LW-S work difference—are most likely to respond and survive in the long term after CRT. However, when septal scarring is present on CMR, this likelihood is reduced. This work adds to the building evidence on the value of imaging LV mechanics and scarring in CRT candidates, which can already be achieved in clinical routine.

## Figures and Tables

**Table 1:** Baseline characteristics.

	All Patients (*n* = 170)	Responders (*n* = 121)	Non-Responders (*n* = 49)
**Clinical characteristics**			
Age (years)	67 ± 10	67 ± 10	65 ± 11 (*)
Male (%)	71	65	83 (*)
BMI (kg/m^2^)	27 ± 5	26 ± 5	27 ± 5
NYHA-class	2.4 ± 0.5	2.3 ± 0.5	2.6 ± 0.5 (*)
Ischemic aetiology (%)	28	22	44 (*)
SBP (mmHg)	124 ± 21	126 ± 20	120 ± 21 (*)
DBP (mmHg)	69 ± 11	71 ± 12	69 ± 10
**Medication**			
Beta-blocker (%)	92	90	92
ACEi/ARB (%)	94	97	90 (*)
Aldosterone antagonists (%)	43	41	46
Diuretics (%)	71	69	77
**ECG criteria**			
QRS morphology: LBBB (%)	87	89	81 (*)
QRS duration (ms)	165 ± 19	166 ± 18	162 ± 19
AFib (%)	6	5	8
Paced (%)	11	7	19 (*)
**Device upgrades (%)**	18	11	35 (*)
**ESC guideline class**			
Class I (%)	87	89	80
Class II (%)	13	11	20
**Implanted CRT**			
CRT-P (%)	14	15	11
CRT-D (%)	86	85	89

ACEi = angiotensin-converting-enzyme inhibitor; ARB = angiotensin-II receptor blocker; BMI = body mass index; CRT-P/-D: cardiac resynchronization therapy -pacemaker/-defibrillator; DBP = diastolic blood pressure; ECG = electrocardiogram; ESC = European Society of Cardiology; NYHA = New York Heart Association; SBP = systolic blood pressure. (*) = *p* < 0.05 vs. responders.

**Table 2:** Echocardiographic characteristics: volumes and systolic function.

	All Patients (*n* = 170)	Responders (*n* = 121)	Non-Responders (*n* = 49)
**Pre-CRT**			
LVEDV (mL)	199 ± 75	192 ± 70	211 ± 85 (*)
LVESV (mL)	140 ± 62	136 ± 58	151 ± 71 (*)
LVEF (%)	31 ± 8	31 ± 7	30 ± 8
GLS (%)	9 ± 3	10 ± 4	8 ± 3 (*)
**12 months follow-up**			
LVEDV (mL)	158 ± 70	139 ± 55 (†)	206 ± 81 (*)
LVESV (mL)	97 ± 59	79 ± 42 (†)	144 ± 69 (*)
LVEF (%)	42 ± 11	46 ± 10 (†)	33 ± 10 (*)
GLS (%)	11 ± 3	14 ± 4 (†)	8 ± 4 (*)

EDV = end-diastolic volume; EF = ejection fraction; ESV = end-systolic volume; GLS = global longitudinal strain; LV = left ventricular; (*) = *p* < 0.05 vs. responders. (†) = *p* < 0.05 vs. Pre-CRT.

**Table 3:** Multivariate linear regression analysis with LV end-systolic volume-reduction as dependent variable.

Regression Variable	B	95%CI	VIF	*p*-Value
**Model 1: SF or ApRock ^(1)^**				
Constant	1.170			
SF or ApRock	−26.589	−34.790 to −18.388	1.161	<0.0001
Ischaemic aetiology	5.335	2.256 to 13.327	1.144	0.036
QRS-morphology	−4.034	−14.147 to 6.078	1.048	0.432
QRS-duration	−0.059	−0.236 to 0.118	1.019	0.511
LVEF	−0.015	−0.459 to 0.429	1.038	0.947
**Model 2: SSI ^(2)^**				
Constant	0.532			
SSI	−2.768	−3.687 to −1.849	1.098	<0.0001
Ischaemic aetiology	12.292	4.774 to 19.810	1.037	0.002
QRS-morphology	−2.151	−12.442 to 8.140	1.075	0.680
QRS-duration	−0.067	−0.247 to 0.113	1.024	0.462
LVEF	−0.087	−0.539 to 0.364	1.061	0.704
**Model 3: LW-S work difference ^(3)^**				
Constant	−13.891			
LW-S work difference	−0.013	−0.017 to −0.008	1.168	<0.0001
Ischaemic aetiology	13.267	5.622 to 20.913	1.033	0.001
QRS-morphology	−1.912	−12.476 to 8.653	1.093	0.721
QRS-duration	−0.102	−0.284 to 0.080	1.013	0.269
LVEF	0.457	−0.012 to 0.926	1.095	0.056

^(1)^: Model R^2^ = 0.27, *n* = 170; ^(2)^: Model R^2^ = 0.26, *n* = 167; ^(3)^: Model R^2^ = 0.24, *n* = 166. CI: confidence interval; VIF: variance inflation factors.

**Table 4:** Multivariate linear regression analysis with LV end-systolic volume-reduction as dependent variables.

Regression Variable	B	VIF	95%CI	*p*-Value
Constant	−35.107			
Anterior scar (%)	0.001	2.921	−0.423 to 0.425	0.999
Septal scar (%)	0.466	3.250	0.010 to 0.921	0.015
Inferior scar (%)	0.028	3.100	−0.370 to 0.426	0.889
Lateral scar (%)	0.198	2.947	−0.199 to 0.595	0.327

*n* = 170. R^2^ = 0.21. Abbreviations in Table 3.

**Table 5:** Multivariate linear regression analysis with LV end-systolic volume-reduction as dependent variables.

Regression Variable	B	95%CI	VIF	*p*-Value
**Model 1: SF or ApRock + septal scarring ^(1)^**				
Constant	−6.639			
SF or ApRock	−25.689	−33.643 to −17.735	1.123	<0.0001
Septal scar (%)	0.315	0.072 to 0.558	1.160	0.011
QRS-morphology	−3.530	−13.504 to 6.443	1.048	0.486
QRS-duration	−0.036	−0.212 to 0.140	1.034	0.686
LVEF	0.065	−0.380 to 0.509	1.067	0.775
**Model 2: SSI + septal scarring ^(2)^**				
Constant	−5.405			
SSI	−2.694	−3.605 to −1.772	1.110	<0.0001
Septal scar	0.413	0.172 to 0.653	1.100	0.001
QRS-morphology	−1.694	−11.958 to 8.570	1.076	0.745
QRS-duration	−0.045	−0.226 to 0.135	1.040	0.622
LVEF	−0.017	−0.473 to 0.440	1.092	0.942
**Model 3: LW-S work difference + septal scarring ^(3)^**				
Constant	−20.610			
LW-S work difference	−0.013	−0.017 to −0.008	1.171	<0.0001
Septal scar	0.460	0.218 to 0.703	1.088	<0.0001
QRS-morphology	−1.288	−11.789 to 9.212	1.095	0.809
QRS-duration	−0.075	−0.258 to 0.107	1.031	0.417
LVEF	0.528	0.057 to 0.998	1.117	0.082

^(1)^: Model R^2^ = 0.30, *n* = 170; ^(2)^: Model R^2^ = 0.27, *n* = 167; ^(3)^: Model R^2^ = 0.27, *n* = 166. Abbreviations in Table 3.

**Table 6:** Predictors of CRT responses in ischemic and non-ischemic heart disease.

	AUC	95%CI	Sensitivity (%)	Specificity (%)
**Ischemic heart disease**				
SF or ApRock + septal scar	0.80	0.67 to 0.93	74	91
SSI + septal scar	0.76	0.63 to 0.90	46	91
LW-S work difference + septal scar	0.75	0.59 to 0.88	47	91
**Non-ischemic heart disease**				
SF or ApRock + septal scar	0.87	0.77 to 0.96	94	64
SSI + septal scar	0.82	0.74 to 0.90	90	63
LW-S work difference + septal scar	0.79	0.68 to 0.90	91	65

CI: confidence interval. Equations in Figure 1.

## Data Availability

The data underlying this article will be shared on reasonable request to the corresponding author.

## Supplementary Materials

The following supporting information can be downloaded at: https://www.mdpi.com/article/10.3390/jcm12186108/s1, Table S1: Frequency of mechanical dyssynchrony before CRT in all study groups; Table S2: Predictors of CRT responses in only those patients with LBBB; Video S1: Echocardiographic four-chamber views of a patient with presence of septal flash and apical rocking prior to CRT implantation. This video corresponds to Case 1. The patient had an LVEF < 35%, LBBB on ECG, and a QRS of 153 ms. No scarring was observed on CMR-LGE. Left panel: status prior to CRT implantation. Note the rapid inward motion of the septum early in systole, followed by the septal-to-lateral rocking motion of the apex. The patient was predicted to be a responder. Right panel: status after 12 months follow-up. Note the resynchronized contraction, with improved LV systolic function and reduced volumes. The patient became a responder; Video S2: Echocardiographic four-chamber views of a patient with absence of septal flash and apical rocking prior to CRT implantation. This video corresponds to Case 2. The patient had an LVEF < 35%, LBBB on ECG, and a QRS of 164 ms. No scarring was observed on CMR-LGE. Left panel: status prior to CRT implantation. Note that no septal flash nor apical rocking is observed. The patient was predicted to be a non-responder. Right panel: status after 12 months follow-up. Note that the LV systolic function and volumes appear unchanged. The patient became a non-responder; Video S3: Echocardiographic four-chamber views of a patient with presence of septal flash and apical rocking prior to CRT implantation. This video corresponds to Case 3. The patient had an LVEF < 35%, LBBB on ECG, and a QRS of 160 ms. Septal scarring was observed on CMR-LGE, in the mid- and apical regions of the septum. Left panel: status prior to CRT implantation. No systolic shortening can be seen in the mid- and apical regions of the septum. Note that the basal septum still shows a septal flash, and that apical rocking can be seen in the apex. The patient was predicted to be a non-responder. Right panel: status after 12 months follow-up. Note that the LV systolic function and volumes appear unchanged. The patient became a non-responder; Video S4: Echocardiographic four-chamber views of a patient with presence of septal flash and apical rocking prior to CRT implantation. This video corresponds to Case 4. The patient had an LVEF < 35%, LBBB on ECG, and a QRS of 160 ms. Inferior wall scarring was observed on CMR-LGE. Left panel: status prior to CRT implantation. Note the septal flash and apical rocking. The patient was predicted to be a responder. The patient was predicted to be a responder. Right panel: status after 12 months follow-up. Note the resynchronized contraction, with improved LV systolic function, and reduced volumes. The patient became a responder.

## Abbreviations

ApRock	apical rocking
CMR	cardiac magnetic resonance imaging
CRT	cardiac resynchronization therapy
GLS	global longitudinal strain
LBBB	left bundle branch block
LGE	late gadolinium enhancement
LV	left ventricular
LVEF	left ventricular ejection fraction
LW-S	lateral-to-septal
SF	septal flash
SSI	systolic stretch index
