# Supplementary material for: Mechanical Dyssynchrony Combined with Septal Scarring Reliably Identifies Responders to Cardiac Resynchronization Therapy
Source: J Clin Med. 2023 Sep 21;12(18):6108. doi: 10.3390/jcm12186108 (PMC10531814; doi:10.3390/jcm12186108)
Supplement: Supplementary file 1 [file jcm-12-06108-s001.zip › jcm-2561527-supplementary/jcm-2561527-supplementary.pdf]

Mechanical dyssynchrony combined with septal scarring reliably identifies responders to cardiac resynchronization therapy

Duchenne et al. Mechanical dyssynchrony and scarring in CRT

SUPPLEMENTS: TABLES

Table S1: Frequency of mechanical dyssynchrony before CRT.

	All patients (n=170)	Responders (n=121)	Non-responders (n=49)
**SF or ApRock (%)**	70	86	30 (*)
**SSI≥3.1% (%)**	69	80	40 (*)
**LW-S≥860 mmHg·% (%)**	69	80	36 (*)

(*) = p<0.05 vs. responders.

Table S2: Predictors of CRT-response in only those patients with LBBB (n=148).

	AUC	95%CI
**Markers**		
SF or ApRock	0.73	0.66 to 0.84
SSI	0.69	0.58 to 0.79
LW-S work difference	0.76	0.65 to 0.86
**Markers + septal scarring**		

SF or ApRock + septal scar	0.85	0.77 to 0.93
SSI + septal scar	0.82	0.74 to 0.89
LW-S work difference + septal scar	0.84	0.75 to 0.92

All p>0.05 vs. the same markers in the entire study cohort (n=170).
